# MiR-30a inhibits BECN1-mediated autophagy in diabetic cataract

**DOI:** 10.18632/oncotarget.20483

**Published:** 2017-08-24

**Authors:** Lu Zhang, Rong Cheng, Yusen Huang

**Affiliations:** ^1^ Department of Ophthalmology, School of Medicine, Shandong University, Jinan 250012, China; ^2^ Qingdao Eye Hospital, Shandong Eye Institute, Shandong Academy of Medical Sciences, Qingdao 266071, China; ^3^ College of Medicine, Qingdao University, Qingdao 266071, China

**Keywords:** miR-30a, BECN1, autophagy, diabetic cataract, lens epithelial cells

## Abstract

**Purpose:**

To investigate the role of microRNAs in the regulation of autophagy and apoptosis in lens epithelial cells (LECs) during diabetic cataract formation.

**Methods:**

A miRNA microarray study and quantitative real-time PCR were performed to identify the expression of miRNAs in LECs of diabetic cataract. Human LECs were cultured in high glucose conditions as a diabetic cataract model. BECN1 and LC3B were detected by Western blotting and quantitative real-time PCR. The extent of apoptosis was measured using FACSCalibur flow cytometry.

**Results:**

Downregulation of miR-30a was identified in LECs attached to diabetic cataract tissues. By the bioinformatic assay and the luciferase activity assay, BECN1 was found to be a direct target of miR-30a. MiR-30a reduced the BECN1-mediated autophagy activity induced by high glucose in LECs *in vitro*. The ratio of LECs apoptosis was also decreased.

**Conclusion:**

MiR-30a was involved in the inhibition of autophagy by targeting BECN1 in LECs in human diabetic cataract.

## INTRODUCTION

Cataracts are a leading cause of blindness and visual impairment in the world [1]. A significant relationship has been revealed between diabetes mellitus (DM) and diabetic cataract as the younger ages and severity of cataracts were found in diabetic patients [2]. One of the reasons may be the high glucose (HG) in such patients. Human lens epithelial cells (LECs) are a single layer of polygonal cuboidal cells that act as the major source of transport, metabolism and detoxification in lens development [3]. The integrity and survival of LECs are critical for lens transparency [4]. However, the molecular mechanism during the process of diabetic cataract formation remains largely unknown.

Macroautophagy (later called autophagy), a multi-step process involving cellular protein and organelle degradation and nutrition recycle, is essential for cell survival and development [5–7]. It plays pathophysiologic roles in many types of cataracts, such as hereditary cataract [8, 9], posterior capsule opacification [10] and age-related cataract [11]. The degradation and clearance of LECs protein aggregation and organelles through an autophagosomal–lysosomal pathway is critically important for maintaining lens transparency.

MicroRNAs (miRNAs) are a group of small RNAs, approximately 21-23 nucleotides in length. They have been identified as non-coding protein and silence gene expression as either mRNA degradation or translational inhibition by targeting the 3’UTR region of mRNA molecules [12, 13]. MiRNAs have been shown to be associated with development, differentiation, maturation, proliferation, apoptosis and autophagy [13–15]. Recently, the function of miRNAs in multiple eye tissues has been disclosed. For example, miRNA-96 was observed to affect the survival and apoptosis of retinal ganglion cells through the activation of caspase-2 [16] and miRNA-100 via the phosphorylation pathway [17]. MiR-182 could target NOX4 directly, thus enhancing neurite outgrowth in isolated trigeminal sensory neurons and recover corneal sensation in hyperglycemic conditions [18]. We previously found that miR-204 inhibited epithelial-mesenchymal transition (EMT) by directly targeting SMAD4 in human posterior capsule opacification [19]. In the present study, we investigated the role of miRNAs in the regulation of autophagy and apoptosis in LECs during diabetic cataract formation.

## RESULTS

### MiR-30a was downregulated in human diabetic cataract

**Figure 1:**
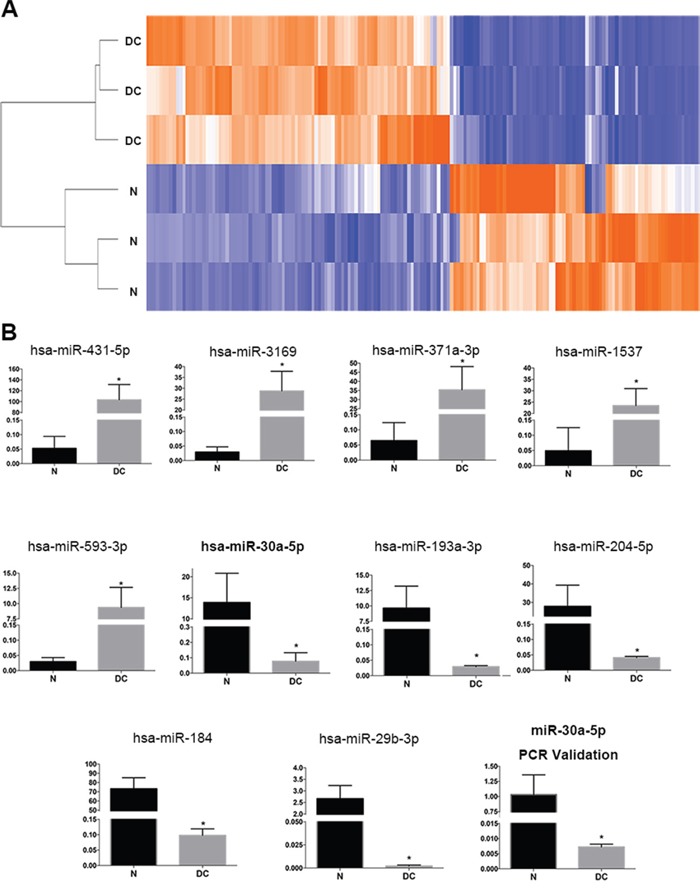
Expression of miRNAs in human diabetic cataract tissues **(A)** The heatmap of three normal lens samples (N) and three diabetic cataract samples (DC). A total of 92 miRNAs were identified to be upregulated more than two folds in diabetic cataract tissues, and 75 miRNAs were identified to be downregulated. **(B)** The expression of the top 5 upregulated miRNAs (miR-431-5p, miR-3169, miR-371a-3p, miR-1537 and miR-593-3p) and top 5 downregulated miRNAs (miR-30a-5p, miR-193a-3p, miR-204-5p, miR-184 and miR-29b-3p) (*P<0.005, N as control). Real-time PCR was performed using nine normal lens tissues and nine diabetic cataract tissues. The relative expression levels of hsa-miR-30a were downregulated significantly in lens epithelial cells attached to diabetic cataract compared with those in normal lens tissues (*P < 0.05, N as control). Significant differences are indicated by t-test (*P < 0.05).

To test the expression of miRNAs in LECs, three donated normal transparent lens tissues and three diabetic cataract tissues were used to perform a miRNA microarray study. The characteristics of the diabetic patients are presented in Table 1. The patient age was 60.67±9.07 years (mean±SD). The duration of DM was 9.00±3.61 years. The blood glucose was 9.47±2.05 mmol/L, and HbA1c (%) was 7.77±1.40. Ninety-two miRNAs were identified to be upregulated more than two folds in diabetic cataract tissues compared with transparent lens tissues (Figure 1A). Meanwhile, 75 miRNAs were identified to be downregulated more than two folds in diabetic cataract tissues compared with transparent lens tissues. The top 5 upregulated miRNAs were miR-431-5p, miR-3169, miR-371a-3p, miR-1537 and miR-593-3p, and the top 5 downregulated miRNAs were miR-30a-5p, miR-193a-3p, miR-204-5p, miR-184 and miR-29b-3p (Figure 1B). As miR-30a is relevant to autophagy [20] and was downregulated more than 180 folds in our miRNA expression profile, we chose it for a further investigation. A quantitative real-time PCR (RT-PCR) was performed to validate the miRNA microarray study and observe the expression of miR-30a. The involved patients, aged 61.89±5.37 years, had a duration of DM of 10.22±6.08 years. The blood glucose was 9.11±2.59 mmol/L, and HbA1c (%) was 8.33±1.51. The expression of miR-30a was downregulated significantly in diabetic cataract tissues compared to transparent lens tissues, which corresponded to the result of the miRNA microarray study.

**Table 1:** Characteristics of the diabetic patients

	Mean±SD			
	Age at surgery (year)	Duration of DM (year)	Blood glucose	HbA1c (%)
Group 1	60.67±9.07	9.00±3.61	9.47±2.05	7.77±1.40
Group 2	61.89±5.37	10.22±6.08	9.11±2.59	8.33±1.51

Group 1: patients cataract tissues used for the miRNA microarray study; Group 2: patients cataract tissues used for the quantitative RT-PCR; DM: diabetes mellitus; HbA1c: glycated hemoglobin.

### MiR-30a directly targeted BECN1

**Figure 2:**
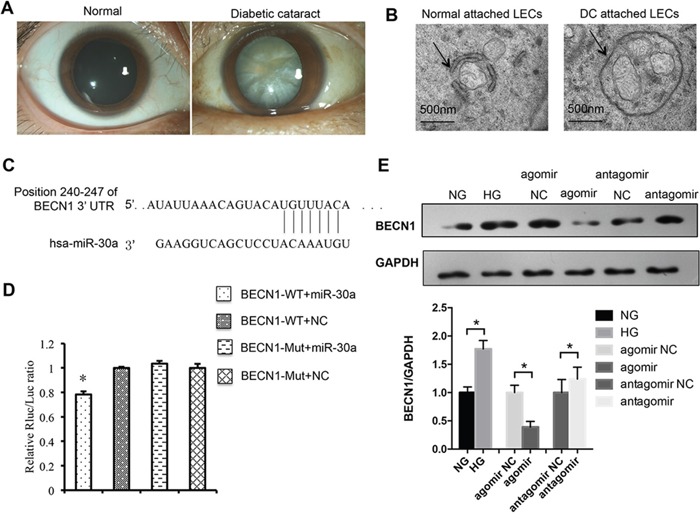
MiR-30a targeted human BECN1 and downregulated BECN1 protein **(A)** Slit lamp photos of a transparent lens vs. an opaque lens in a patient with diabetic cataract. **(B)** Electron micrographs of double membrane autophagosomes in lens epithelial cells (LECs). The autophagosomes encapsulated several mitochondria in LECs of diabetic cataract and were larger than those normal ones. **(C)** Position of the miR-30a targeted sequence in the 3’-UTR of BECN1 mRNA. **(D)** In a dual luciferase reporter assay, the relative luciferase activity decreased significantly when pmiR-RB-REPORT-BECN1-3’UTR and miR-30a agomir were cotransfected (agomir NC as control, *P<0.05). The mutation of the perfectly complementary sites in the BECN1-3’UTR abolished the suppressive effect of miR-30a on BECN1. **(E)** Western blot analysis. High glucose induced the protein expression of BECN1 in LECs (*P<0.05, NG as control). BECN1 expression was downregulated when LECs were transfected with miR-30a agomir and upregulated with miR-30a antagomir (*P<0.05, agomir NC and antagomir NC as control). Significant differences are indicated by t-test (*P < 0.05).

Lens slit lamp photos are presented in Figure 2A. Transmission electron microscopy was performed, and double membrane autophagosomes were detectable not only in LECs attached to normal tissues but also those in diabetic cataract tissues (Figure 2B). However, the single autophagosome in LECs of diabetic cataract tissues was larger than that in normal LECs. Moreover, multiple mitochondria were encapsulated in one autophagosome in LECs attached to diabetic cataract tissues, which illustrated the inverted autophagy activity induced by high blood glucose in patients with diabetes.

From a bioinformatics database (TargetScanHuman, http://www.targetscan.org/vert_70/) we found that miR-30a could target BECN1 3’UTR, an important regulator of autophagy, at positions of 240-247 (Figure 2C). MiR-30a agomir could overexpress miR-30a, while miR-30a antagomir could inhibit the expression of miR-30a. A dual luciferase reporter assay was performed to identify the direct relationship of miR-30a and BECN1. The relative luciferase activity was observed to decrease significantly when the transfected miR-30a agomir and the 3’UTR mutant of BECN1 abolished the suppressive effect of miR-30a on BECN1 (Figure 2D). Western blotting was also performed, and the protein expression of BECN1 was found to be decreased by miR-30a agomir in LECs in the presence of HG and be increased by miR-30a antagomir when compared with the negative control (NC) (Figure 2E). The mRNA expression was not affected by miR-30a agomir or antagomir (data not shown). All the results revealed the repression of miR-30a on BECN1 at the translational level. Because BECN1 is a key regulator of autophagy, we next investigated the regulation of miR-30a on BECN1-mediated autophagy activity in LECs.

### MiR-30a repressed autophagy in LECs

**Figure 3:**
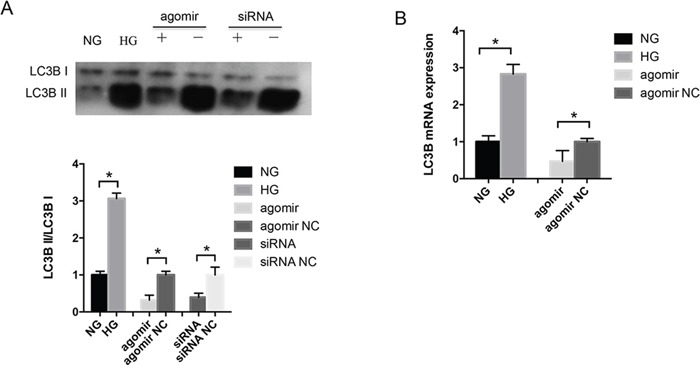
MiR-30a regulated autophagy in lens epithelial cells (LECs) **(A)** Western blot analysis. In high glucose, the ratio of LC3B II/LC3B I was elevated compared with in normal glucose (*P<0.05). MiR-30a agomir downregulated the ratio directly (agomir NC as control, *P<0.05); BECN1 siRNA also repressed LC3B II expression compared to siRNA NC (P*<0.05). **(B)** The level of LC3B mRNA expression showed an increase in LECs due to high glucose and a decrease when LECs were treated with miR-30a agomir (agomir NC as control, *P<0.05). Significant differences are indicated by t-test (*P < 0.05).

LECs were exposed to 5 mM D-glucose (normal glucose) as a control group and 30 mM D-glucose (HG) as an experiment group, respectively, for 48 hours, and then treated with miR-30a agomir and agomir NC directly for 24 hours. In the HG environment, the ratio of LC3B II and LC3B I proteins increased significantly in LECs; the expression was decreased when LECs were transfected with miR-30a agomir compared with agomir NC (Figure 3A). As LC3B II was measured as an autophagosome-associated marker [21], the increased ratio of LC3B II/LC3B I represented the active autophagy activity. We also employed BECN1 siRNA to knock down the gene endogenous expression and found that BECN1 siRNA decreased the ratio of LC3B II/LC3B I. The increased LC3B mRNA expression was induced by HG, and miR-30a agomir inhibited the HG-induced LC3B expression (Figure 3B). The above findings indicated that HG stimulated the autophagy activity in LECs, and miR-30a repressed the level of autophagy by modulating BECN1 expression.

### MiR-30a decreased apoptosis of LECs in an HG condition

**Figure 4:**
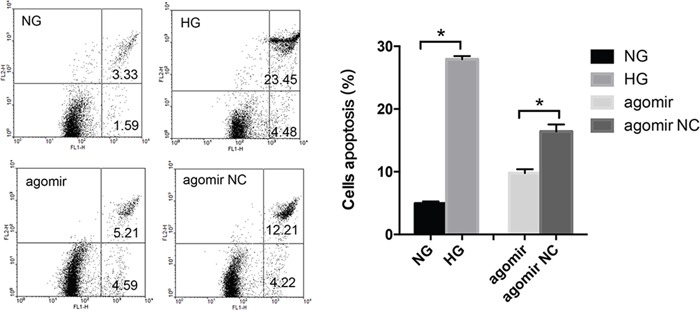
MiR-30a influenced high glucose-induced lens epithelial cells (LECs) apoptosis Lower left: survivals; lower right: early apoptosis; upper right: post-apoptotic necrosis. The apoptosis rate of LECs was from 4.94% to 27.93% in the presence of high glucose (*P<0.05, NG as control); the rate decreased from 16.42% to 49.8% when LECs were treated with miR-30a agomir (*P<0.05, agomir NC as control). Significant differences are indicated by t-test (*P < 0.05).

As both autophagy and apoptosis are involved in cell death, we tried to investigate whether miR-30a could regulate the LECs apoptosis. In apoptotic cells, FITC could conjugate annexin V and invert phosphatidylserine. By flow cytometry, we observed that LECs apoptosis was induced by HG (Figure 4). The rate of HG-induced apoptosis in LECs increased from 4.94% to 27.93%. Meanwhile, the rate of LECs apoptosis decreased from 16.42% to 9.8% when LECs were treated with miR-30a agomir compared to agomir NC. These results revealed that miR-30a might help protect LECs from apoptosis in an HG condition.

## DISCUSSION

Since the function of miRNAs in LECs during diabetic cataract formation was not well unknown, we performed a miRNA microarray study using normal transparent lens tissues and diabetic cataract tissues. MiR-30a was chosen for a further study because it was reported to be related with autophagy, an intracellular self-defense mechanism for sustaining of metabolic homoeostasis [22–24]. There were some reports on diabetic cataract using HG conditions *in vitro* [25–27]. Du et al.[27] set 50 mmol/L glucose as HG conditions in treating rat lenses, finding that all the lenses in the normal glucose group (5.56 mmol/L glucose) appeared clear while most lenses in the HG group were covered with opaque rings after 5 days. In our previous study [28], human lens capsular bags were maintained in 5 mM D-glucose or 25 mM D-glucose as a diabetic cataract model *in vitro*, which adequately recapitulated the diabetic state *in vivo*. Thus, we chose human lens epithelial cells cultured in HG conditions as the diabetic cataract model in the present study. We identified that miR-30a was downregulated in LECs attached to diabetic cataract tissues; meanwhile, miR-30a could decrease the levels of HG-induced autophagy by targeting BECN1 in LECs besides inhibiting LECs apoptosis in HG.

miRNAs are involved in almost every aspect of cellular processes like differentiation, proliferation, apoptosis, metabolism and environmental stress [13], so many diseases have been investigated to be associated with miRNA expression [29]. miRNAs have a great relationship with the lens development and cataract formation. However, there has been no report about the effect of miR-30a on LECs during cataract formation. Studies of miR-30a have been focused mainly on diseases like cancers, ischemic injury and cardiac pathology [24, 30, 31]. In age-related cataract, miR-15a-5p, miR-15a-3p and miR-16-1-5p were reported to increase in LECs and regulate apoptosis by suppressing the expression of the anti-apoptotic genes [32]. MiR-34a was highly expressed in unclear, cortical and posterior subcapsular cataract tissues and was correlated with lens opacity severity [33]. Moreover, miR-34a could enhance the apoptosis by downregulating Bcl-2 and SIRT1 in human LECs [34]. Let-7b is correlated with age-related cataract and can enhance the ultraviolet irradiation-induced apoptosis of LECs [35]. The increase of let-7b may represent a risk factor in the formation of age-related cataracts [36]. Although miR-29a and miRNA-29c might target the BCL2-modifying factor to regulate apoptosis levels induced by HG in the LECs of diabetic rats [37], the contributions of miRNAs to the human diabetic cataract pathogenesis are not clearly understood.

BECN1 is involved in multiple cellular processes and is thought to be a key modulator of cellular autophagy through mediating vesicle-trafficking processes. It has been identified as a direct target of miR-30a mostly in cancers and ischemic injury [38, 39]. In this study, we identified that HG induced the increase of BECN1 in LECs and did help miR-30a to regulate autophagy activity in LECs. By transmission electron microscopy, larger double membrane autophagosomes were shown encapsulate several mitochondria in LECs attached to diabetic cataract tissues compared with those in normal lens tissues. The ratio of LC3B II, a marker related to autophagosomes [21], and LC3B I revealed the repressing regulation of miR-30a on autophagy through BECN1. While excess autophagy has been implicated in type II cell death, apoptosis is described as a physiological cell death pathway. Apoptosis is commonly necessary in the differentiation and development [40, 41]. A few reports have demonstrated the complex interaction between autophagy and apoptosis [42–45]. Autophagy was observed to promote apoptosis via the Akt/mTOR signaling pathway in breast cancer cell lines during the combined treatment [46]. Moreover, miR-30a and miR-205 were reported to simultaneously suppress TP53INP1 expression to mediate apoptosis after irradiation [22]. In our study, miR-30a depressed LECs apoptosis induced by HG. However, the relationship of autophagy and apoptosis and the mechanism that miR-30a decreases the levels of apoptosis responding to HG in the process of diabetic cataract formation require further investigations.

## MATERIALS AND METHODS

### Reagents and chemicals

All miR-30a agomir, agomir NC, antagomir, antagomir NC and BECN1 siRNA were purchased from RiboBio (Guangzhou, China). Anti-LC3B antibody was obtained from Invitrogen (Frederick, MD, USA), anti-BECN1 from Abcam (Cambridge, MA, USA), and anti-GAPDH from Kangchen Bio-tech (Shanghai, China).

### Lens tissue sample collection and cell culture

This study was performed following the tenets of the Declaration of Helsinki and was approved by the ethics committee of Shandong Eye Institute. Diabetic cataract tissues were collected at surgery (YH). Normal lens tissues were provided by the eye bank of Shandong Eye Institute.

Human LECs-B3 were maintained in DMEM/F12 with 10% fetal bovine serum, 1% penicillin G and 1% streptomycin containing 5 mM D-glucose (normal glucose) and 30 mM D-glucose (HG), respectively, in a humidified 5% CO_2_ incubator at 37°C. After being cultured for 48 hours, LECs were transfected with 200 nmol/L miR-30a agomir, agomir NC, antagomir and antagomir NC, respectively, for another 24 hours according to the manufacturer's protocol. BECN1 si-RNA was transfected using lipofectamine^TM^ 2000 (Invitrogen) for 48 hours. Subsequently, cells were washed twice with cold phosphate-buffered saline and collected for further analysis. The sequences of BECN1 siRNA were as follows: 5’-CAGUUUGGCACAAUCAAUA-3’.

### MiRNA microarray

The miRNA microarray study was performed at Kangchen Bio-tech using a miRCURYTM LNA array (v.18.0, Exiqon, Vedbaek, Denmark). All the RNAs were isolated using TRIzol (Invitrogen) and a miRNeasy mini kit (QIAGEN, Hilden, Germany) according to the manufacturers’ instructions. After RNA isolation from the samples, the miRCURY™ Hy3™/Hy5™ Power labeling kit (Exiqon) was used following the manufacturer's guideline for miRNA labelling. Next, the Hy3TM-labeled samples were hybridized on the miRCURYTM LNA array (v.18.0, Exiqon) according to the array manual. Scanned images were then imported into GenePix Pro 6.0 software (Axon) for grid alignment and data extraction. The heatmap was performed using Cluster 3.0 and TreeView 1.1.6r4.

### Quantitative RT-PCR

Total RNA was isolated with a miRNeasy mini kit (QIAGEN) according to the manufacturer's protocol. The cDNA of miR-30a and U6 were synthesized with a miRNA first-strand synthesis kit (Clontech, Dalian, China), and the cDNAs of BECNI and LC3B were synthesized with an MMLV first-strand synthesis kit (Invitrogen). The SYBR Green (Clontech) was used on an ABI 7500 system (Applied Biosystems, Foster City, CA, USA). GAPDH and U6 were used to normalize the expression level. All reactions were performed in triplicate. The 2 -ΔΔCt method was utilized to calculate the expression of each mRNA. The primer sequences employed were as follows: LC3B F’CCTTGTACCTGACCATGTCAACA, R’CCTGGGAGGCATAGACCATGTA.BECN1.

F’ TGTCCACAGAAAGTGCCAACA, R’ CCTCA CAGAGTGGGTGATCCA. The primers of miR-30a and U6 were purchased from Takara (Dalian, China) without sequence information.

### Luciferase activity assay

The 3’UTR of BECN1 containing the putative target site for miR-30a and the mutant sequences were amplified by PCR and inserted into the pmiR-RB-REPORT (RiboBio). By employing Lipofectamine 2000 (Invitrogen), the cells were transiently transfected with the wild-type or mutant reporter plasmid, miRNA agomir or agomir NC for 48 hours. Subsequently, luciferase activity was measured with the Dual-Luciferase Assay-System (Promega). All reactions were tested in triplicate.

### Transmission electron microscopy

Normal human lenses and diabetic cataract tissues were fixed with 2.5% glutaraldehyde for 4 hours or longer and then with 1% osmic acid for 1 to 1.5 hours. The tissues were dehydrated in 50%, 70%, 90% and 100% acetone three times, each for 15 minutes, before embedded in epoxy resin (EMS, Epon 812, 14120). Sections of 70 nm in thickness were cut (Reichert-Jung ULTRACUT) and collected with a copper net. Thin sections were stained with uranyl acetate and lead citrate, each for 15 minutes, and viewed under a transmission electron microscope (JEM1200).

### Western blot analysis

The samples were homogenized in buffer containing RIPA and phenylmethylsulfonyl fluoride. The homogenates were assayed on polyacrylamide gels, transferred onto PVDF membranes (Thermo Fisher Scientific, Billerica, USA) and then probed with specific primary antibodies.

### Assessment of apoptotic cells

The extent of apoptosis was measured through a sannexinV-FITC apoptosis detection kit (Beyotime Institute of Biotechnology, Hangzhou, China) as described in the manufacturer's instruction. The cells were gently resuspended in annexin-V binding buffer and incubated with annexinV-FITC/PI in the dark for 15 minutes. The samples were analyzed using FACSCalibur flow cytometry (BD Bioscience). The fraction of cell population in different quadrants was analyzed using quadrant statistics. Cells in the lower left quadrant represented survivals, in the lower right quadrant represented early apoptosis, and in the upper right quadrant represented post-apoptotic necrosis.

### Statistical analysis

All experiments were performed three times. All results are expressed as means±standard deviation (SD) unless indicated otherwise. The analysis of differential expression was performed using SPSS software (Version 16.0, Chicago, IL, USA) and GraphPad Prism software 5.0 (GraphPad Software). A P value of < 0.05 was considered statistically significant.

## CONCLUSIONS

Our study provides important insights into the role of miR-30a in the suppression of autophagy by targeting BECN1 in LECs and addresses the role of miR-30a in LECs in human diabetic cataract. Further studies are needed to focus on the potential development of miR-30a as a new therapeutic agent for diabetic cataract.
